# Trends of Utilization of Systemic Therapies for Metastatic Renal Cell Carcinoma in the Canadian Health Care System

**DOI:** 10.1200/GO.23.00271

**Published:** 2023-11-22

**Authors:** Luisa M. Cardenas, Sunita Ghosh, Antonio Finelli, Lori Wood, Christian Kollmannsberger, Naveen Basappa, Jeffrey Graham, Daniel Heng, Georg Bjarnason, Denis Soulières, Dominick Bossé, Vincent Castonguay, Ramy Saleh, Simon Tanguay, Bimal Bhindi, Rodney H. Breau, Frederic Pouliot, Aly-Khan A. Lalani

**Affiliations:** ^1^ Department of Oncology, Juravinski Cancer Centre, McMaster University, Hamilton, ON, Canada; ^2^ Department of Medical Oncology, Faculty of Medicine and Dentistry, University of Alberta, AB, Canada; ^3^ Division of Urology, University Health Network, Toronto, ON, Canada; ^4^ Department of Medicine and Urology, Dalhousie University, Halifax, NS, Canada; ^5^ Department of Medical Oncology, BC Cancer Agency, Vancouver, BC, Canada; ^6^ Department of Medical Oncology, Cross Cancer Institute, Edmonton, AB, Canada; ^7^ Cancer Care Manitoba Research Institute, University of Manitoba, Winnipeg, MB, Canada; ^8^ Department of Oncology, Tom Baker Cancer Centre, Calgary, AB, Canada; ^9^ Department of Medical Oncology, Sunnybrook Health Sciences Centre—Odette Cancer Centre, Toronto, ON, Canada; ^10^ Hematology-Oncology Department, CHUM—Centre Hospitalier de l’Université de Montréal, Montreal, QC, Canada; ^11^ Medical Oncology Division, The Ottawa Hospital Regional Cancer Centre, Ottawa, ON, Canada; ^12^ Hematology-Oncology Department, Centre Hospitalier Universitaire Pavillon l'Hôtel-Dieu de Quebec, Quebec City, QC, Canada; ^13^ Department of Medical Oncology, McGill University, Montreal, QC, Canada; ^14^ Division of Urology, McGill University and McGill University Health Centre, Montreal, QC, Canada; ^15^ Department of Surgery, Section of Urology, University of Calgary, Calgary, AB, Canada; ^16^ Department of Surgery, The Ottawa Hospital Research Institute, University of Ottawa, Ottawa, ON, Canada; ^17^ Department of Urology, CHU de Quebec, Université Laval, Quebec City, QC, Canada

## Abstract

**PURPOSE:**

Standard-of-care therapies for metastatic renal cell carcinoma (mRCC) have greatly evolved. However, the availability of emerging options in global health care systems can vary. We sought to describe the integration and usage of systemic therapies for mRCC in Canada since 2011.

**METHODS:**

We included patients with mRCC enrolled in the Canadian Kidney Cancer Information System, a prospective cohort of patients from 14 Canadian academic centers, who received systemic therapy from January 1, 2011, to December 31, 2021. Patients were stratified by treatment era (cohort 1: 2011-2015, cohort 2: 2016-2021). Stacked bar charts were used to present treatment proportions; Sankey diagrams were used to show the evolution of treatment sequencing between the two cohorts.

**RESULTS:**

Four thousand one hundred seven patients were diagnosed with mRCC, of whom 2,752 (67%) received systemic therapy. Among these patients, mean age was 64 years, 74% were male, 75% had clear cell histology, and International Metastatic RCC Database Consortium risk classification was favorable, intermediate, and poor in 16%, 56%, and 28%, respectively. Utilization of immune checkpoint inhibition (ICI)–based treatments has increased in Canada and reflects global and local patterns of approval and adoption. The use of therapies after doublet ICI has mostly shifted toward vascular endothelial growth factor-tyrosine kinase inhibitors (VEGF-TKIs) that were previously used in first line with subsequent treatments reflecting approved and available agents after previous VEGF-TKI. Clinical trial participation among patients who received systemic therapy was 18% in first, 21% in second, and 24% in third line.

**CONCLUSION:**

In Canada's publicly funded health care system, availability of standard mRCC therapies broadly reflects access from government-funded clinical trials and compassionate access program sources. In an evolving therapeutic landscape, ongoing advocacy is required to continue to facilitate patient access to efficacious therapies.

## Teaser

Access to mRCC therapies in Canada leverages government-funded clinical trials and compassionate program sources.

## INTRODUCTION

Kidney cancer is the 14th most common cancer worldwide, with renal cell carcinoma representing over 90% of cases.^1^ In Canada, there were an estimated 8,100 new cases of kidney cancer and 1,950 related deaths in 2022, making it the eighth most common malignancy.^2^ Over the past decade, important progress has been made in the therapeutic landscape of metastatic renal cell carcinoma (mRCC), characterized by a transition from cytokines, to vascular endothelial growth factor-tyrosine kinase inhibitors (VEGF-TKIs) and more recently to immune checkpoint inhibition (ICI)–based treatments.^3^ Multiple combinations of anti–PD(L)-1 plus either anti–CTLA-4 antibodies (immuno-oncology–based therapy [IO]/IO) or VEGF-TKI (IO/TKI) are now Health Canada approved after demonstrating improved overall survival (OS) compared with previous first-line standard VEGF-TKI.^4-8^ Given the emergence of new treatments, the Kidney Cancer Research Network of Canada produces a regular consensus update on the management of advanced kidney cancer, most recently recommending ICI-based regimens as preferred treatment options in the first-line setting.^9^ Access to modern anticancer therapies in Canada is often challenged by lengthy drug approval and public reimbursement processes. Once approved by Health Canada, new agents undergo review by the Canadian Agency for Drugs and Technologies in Health (CADTH), which makes funding recommendations to provinces and territories. It is ultimately a provincial decision to provide public finding for new drugs, which may lead to disparate access to novel therapies across the country.^10^ Although new drugs undergo provincial reimbursement review, pharmaceutical companies sometimes offer access to patient support programs where new therapies can be accessed in a timelier manner.

**CONTEXT**

**Key Objective**
Over the past decade, the standard of care of metastatic renal cell carcinoma (mRCC) has evolved. However, the availability of novel therapies in global health care systems varies and can be delayed by complex drug approval and reimbursement processes. How have emerging therapies for mRCC been implemented in the Canadian public health care system?
**Knowledge Generated**
The utilization of immune checkpoint inhibition–based therapies in the upfront setting has increased in Canada and reflects global patterns of approval and adoption. Subsequent therapies have largely shifted toward vascular endothelial growth factor inhibitors that were previously used in first line.
**Relevance**
Availability of standard mRCC therapies in Canada leverage government funded, clinical trials, and compassionate drug access programs. Ongoing partnership with patient advocacy groups is important to continue to facilitate patient access to emerging efficacious therapies.

There are limited data on the use of recommended contemporary therapies in routine clinical practice in Canada. The objective of this study is to describe the evolution of systemic therapy use for mRCC in the real-world setting using a prospective Canadian cohort of patients (Canadian Kidney Cancer Information System [CKCis]).

## METHODS

We used the CKCis cohort to identify adults with mRCC who received systemic therapy or were on active surveillance (AS) between January 1, 2011, and December 31, 2021. The CKCis is a national registry that includes patients from 14 academic centers across Canada and is representative of the entire Canadian kidney cancer population.^11^ All sites have Research Ethics Board approval.

Baseline demographic and tumor characteristics were summarized, including the International Metastatic RCC Database Consortium (IMDC) score. This is a prognostic tool that uses six clinical and laboratory risk factors (anemia, thrombocytosis, neutrophilia, hypercalcemia, poor performance status, and time from diagnosis to start of systemic therapy of less than 1 year) to classify patients into favorable-risk (score of 0), intermediate-risk (score of 1-2) and poor-risk (score of 3-6) disease.^12^ Patients were stratified by treatment era (2011-2015 and 2016-2021) and type of therapy received. The year 2016 was chosen as the cutoff date for the two treatment eras, since it corresponds to the approval of the first anti–PD-1, nivolumab, by Health Canada, for previously treated mRCC in 2016,^13^ after it demonstrated superiority against everolimus.^14^ Descriptive statistics were used: mean (standard deviation) for continuous variables and frequency (%) for categorical values. Stacked bar graphs were used to show annual treatment proportions. The patient population was stratified into two cohorts on the basis of year of treatment initiation (cohort 1, 2011-2015; and cohort 2, 2016-2021), and Sankey diagrams were used to show the differences in treatment sequencing during these two eras. SAS version 9.4 (SAS Institute Inc, Cary, NC) was used for the descriptive analysis. The SankeyMATIC tool was used to create the Sankey diagrams.^15^

## RESULTS

From January 1, 2011, to December 31, 2021, 4,107 patients were diagnosed with mRCC, of whom 2,752 (67%) received systemic therapy. Baseline characteristics are shown in Table 1. Among patients who started first-line systemic therapy and had complete clinical data, the median age was 64 years, 74% of were male, 64% were Caucasian, and 75% had clear cell histology. The majority of patients had IMDC intermediate-risk disease (n = 1,221; 56%) and n = 2,098 (76%) had undergone a previous nephrectomy. Rates of upfront cytoreductive nephrectomy were higher in cohort 1 at 29% compared with 23% in cohort 2.

**TABLE 1:**
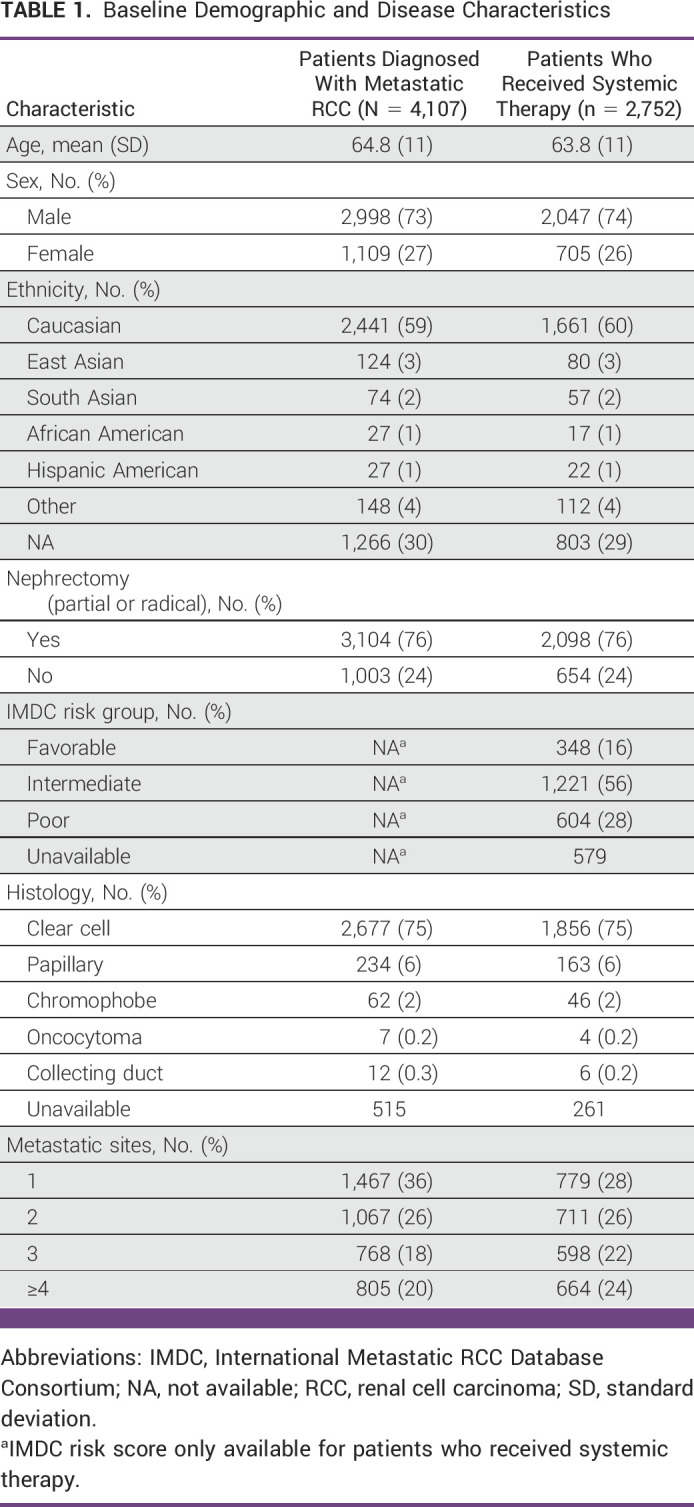
Baseline Demographic and Disease Characteristics

**FIG 1:**
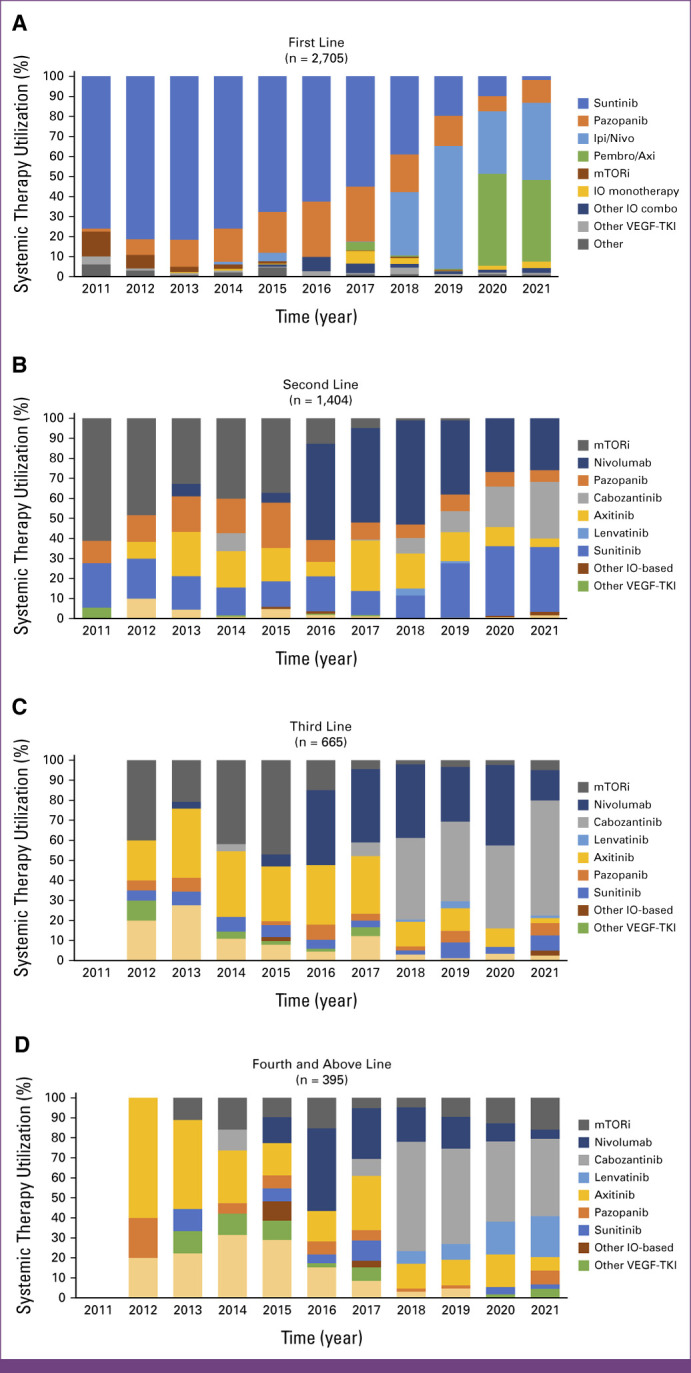
Systemic treatment utilization from 2011 to 2021 by line of therapy: (A) first line, (B) second line, (C) third line, and (D) fourth line and beyond. Bevacizumab (monoclonal antibody targeting the VEGF receptor) was also included in this category. IO monotherapy, atezolizumab, ipilimumab, nivolumab, pembrolizumab; Ipi/Nivo, ipilimumab plus nivolumab; mTORi, mammalian target of rapamycin inhibitor; Other IO-based, other immune checkpoint blockade–based treatment (monotherapy with atezolizumab, ipilimumab, pembrolizumab); Other IO combo, atezolizumab + bevacizumab, avelumab + axitinib, cabozantinib + nivolumab, cabozantinib + ipilimumab, pembrolizumab + lenvatinib; Other VEGF-TKI, other VEGF-tyrosine kinase inhibitor (first line: axitinib, cabozantinib, sorafenib, tivozanib; Pembro/Axi, pembrolizumab plus axitinib; second line and beyond: sorafenib, regorafenib, tivozanib); VEGF, vascular endothelial growth factor.

The use of systemic therapy and change in treatment lines from 2011 to 2021 are depicted in Figure 1. Patients were stratified into year of treatment initiation and line of therapy: first (Fig 1A), second (Fig 1B), third (Fig 1C), and fourth and above (Fig 1D). Therapies are displayed as the names of most commonly used drugs in the respective line of therapy, or grouped for less commonly used agents (ie, other VEGF-TKI, other IO, mammalian target of rapamycin [mTOR] inhibitors, and others). In the first-line setting, VEGF-TKI monotherapy was the most frequent strategy from 2011 to 2017, with sunitinib and pazopanib representing approximately 70% and 20%, respectively, of all therapies used during that period. From 2018 to 2021, the use of first-line VEGF-TKI declined to approximately 35%, replaced by first-line IO/IO (41%) and IO/TKI (21%). In the second-line setting, mTOR inhibitors were the most used agents (40%) from 2011 to 2015. Nivolumab (anti–PD-1 antibody) became a popular second-line choice in 2016 (49%), but its use declined after 2018 when it was approved as a combination therapy with ipilimumab (anti–CTLA-4 antibody) in the first-line setting. Since 2020, second-line VEGF-TKIs have become the most used agents, particularly sunitinib (34%) and cabozantinib (24%). A similar trend was seen in third line, with the usage of mTOR inhibitors decreasing and cabozantinib increasing since 2018 (44%). Overall, clinical trial participation among patients who received systemic therapy was 18% in first line, 21% in second line, and 24% in third line.

**FIG 2:**
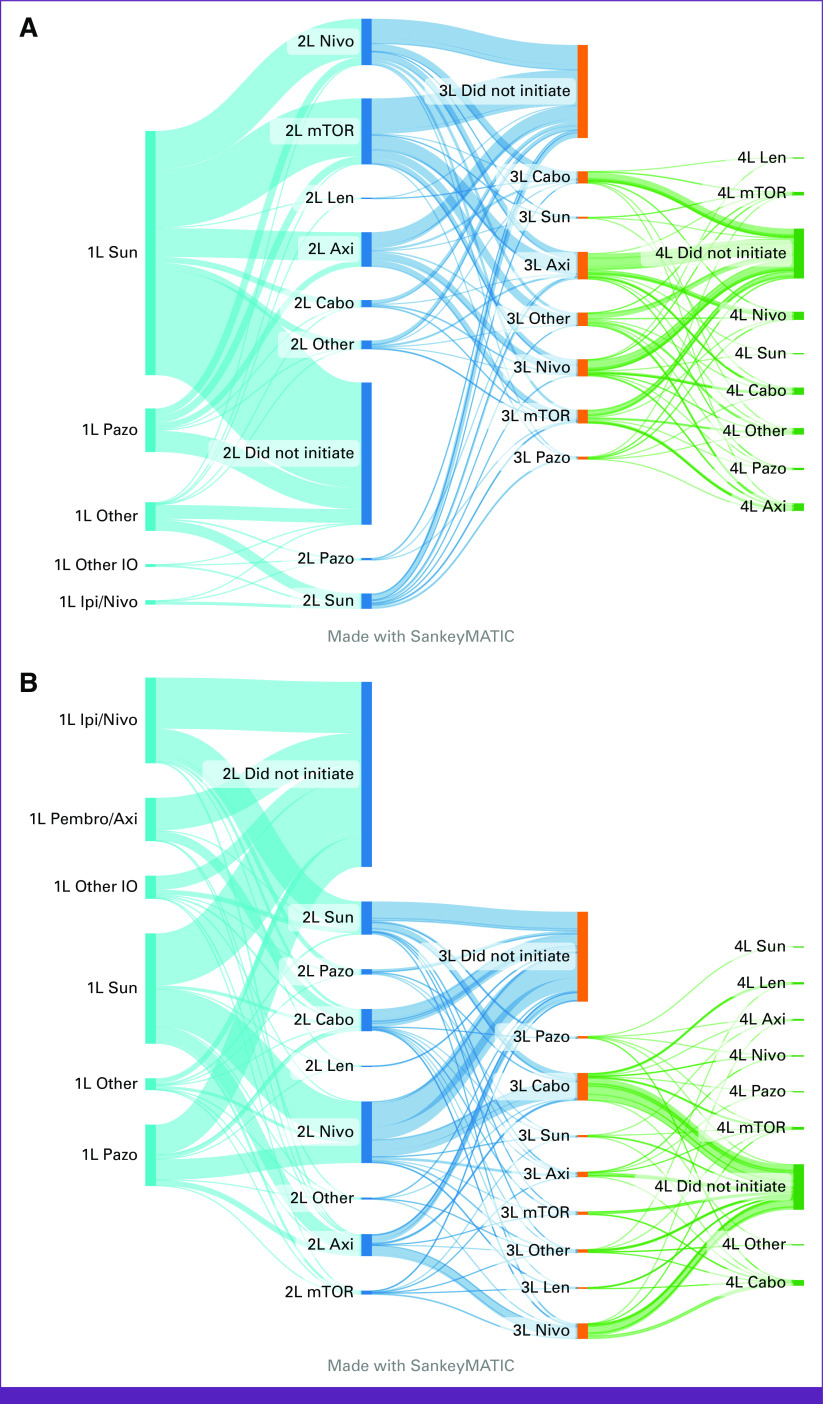
Systemic treatment sequencing from first to fourth line, on the basis of year of systemic therapy initiation. (A) Cohort 1: 2011-2015 (n = 1,040) and (B) cohort 2: 2016-2021 (n = 1,712). 1L, first line; 2L, second line; 3L, third line; 4L, fourth line; Axi, axitinib; Cabo, cabozantinib; Ipi/Nivo, ipilimumab plus nivolumab; Len, lenvatinib; mTOR, mammalian target of rapamycin; Nivo, nivolumab; Other IO, other immune checkpoint blockade–based treatment; Pazo, pazopanib; Pembro/Axi, pembrolizumab plus axitinib; Sun, sunitinib.

The evolution of systemic treatment sequencing from first to fourth line is illustrated in Figure 2. Patients were stratified into two cohorts: cohort 1 shows the treatment sequencing of patients treated between 2011 and 2015 (Fig 2A), and cohort 2 demonstrates the sequence of therapies between 2016 and 2021 (Fig 2B). In cohort 1, the most frequently used second-line therapies after sunitinib were VEGF-TKIs, mTOR inhibitors, and nivolumab. Cohort 2 commonly received sunitinib after first-line IO/IO (ipilimumab plus nivolumab), and cabozantinib after IO/TKI (pembrolizumab [anti–PD-1 antibody] plus axitinib [VEGF-TKI]). A proportion of patients who received first-line treatment did not initiate subsequent line therapy, due to either ongoing response to first line treatment, or inability to receive subsequent line therapy.

## DISCUSSION

The treatment landscape of mRCC has greatly evolved over the past 20 years. More recently, ICI-based combinations have become standard first-line options with an OS benefit beyond previous benchmarks. Although timely access to novel therapies in the Canadian public health care system can be complicated by extensive drug approval and reimbursement processes, our study shows that the trends of utilization of systemic treatments for mRCC in Canada largely reflect global patterns of use. Timely access is due to multiple factors, including patient and physician advocacy, other methods of drug access (including compassionate access offered by pharmaceutical companies), and increasing accessibility to clinical trials.

Although several therapies for mRCC have been approved by Health Canada, a significant process follows from regulatory approval to ultimately having treatments routinely accessible for patients. The first step before a new drug enters the Canadian market is for Health Canada to review a new drug submission. The process can take as long as 300 days. A priority review can be requested for certain qualifying drugs, in which case the process can take up to 180 days.^16^ Once a drug is approved by Health Canada, it can be legally sold and used but it is not automatically publicly reimbursed. Each new oncology drug needs to undergo a reimbursement review process by the CADTH pan-Canadian Oncology Drug Review (pCODR), which performs an objective assessment of the scientific evidence, patient and clinician factors, and economical implications.^17^ CADTH reviews take approximately 150 business days.^18^ The pCODR provides a reimbursement recommendation to all the Canadian provinces and territories, except Quebec, which undergoes a similar process through the Institut national d'excellence en santé et en services sociaux. It is ultimately up to each province or territory to decide whether a drug is reimbursed, considering not only the pCODR recommendations, but also their unique economic resources.^16^ Reimbursement criteria and approval timelines are variable between provinces and territories, leading to significant inequalities in access to cancer therapies across Canada.^16^ A recent study showed remarkable disparities in the reimbursement process for mRCC treatments among Canadian provinces, with a median lag between first and last provincial approval of 20.5 months.^10^

Despite the long process of drug approval and public funding, our study shows that uptake of novel therapies in Canada has generally followed shortly after publication of landmark clinical trials. In 2018, the first ICI combination to be approved by Health Canada in the first-line setting was ipilimumab plus nivolumab after the CheckMate 214 trial demonstrated an OS advantage compared with sunitinib among patients with IMDC intermediate- or poor-risk disease (median OS, 48.1 months *v* 26.6 months; hazard ratio [HR], 0.65 [0.54-0.78]).^4,19^ In 2020, pembrolizumab plus axitinib was Health Canada approved, regardless of IMDC risk score, after OS superiority was demonstrated against sunitinib in the KEYNOTE-426 trial. Median OS was 45.7 months versus 40.1 months (HR, 0.73; *P* < .001).^20^ Two other ICI-based combinations for the first-line treatment of mRCC have since received Health Canada approval and are currently undergoing reimbursement review by CADTH and/or provincial negotiation at the time of this writing. Pembrolizumab plus lenvatinib (VEGF-TKI) has demonstrated an OS benefit compared with sunitinib in the CLEAR study (OS rate at 24 months, 80.2% *v* 69.7%; HR, 0.72; 95% CI, 0.55 to 0.93; median OS not reached).^21^ Nivolumab plus cabozantinib was evaluated in the CheckMate 9ER study, where it also showed improved OS compared with sunitinib (median OS, 37.7 *v* 34.3; HR, 0.70; 95% CI, 0.55 to 0.90).^22^

First-line treatment selection is therefore largely based on disease stratification using the IMDC prognostic criteria, with IO/IO therapy being funded only for patients with IMDC intermediate- and poor-risk disease, whereas IO/TKI is available regardless of IMDC risk status.^9^ For patients who are not candidates for combination-based therapies because of comorbidities or personal preference, both sunitinib and pazopanib remain suitable options. It is recommended to individualize the dose and schedule of these agents on the basis of toxicity.^9^ Figure 1A shows the change toward first-line ICI-based therapies since 2018, with ongoing use of VEGF-TKIs in a minority of patients. AS is considered reasonable for highly selected patients with IMDC favorable-risk, low-volume disease who are asymptomatic and for whom the risks of therapy outweigh its benefits.^9^ A recent analysis of CKCis data revealed that AS is a safe option in a subset of patients.^23^ In our study, 33% of patients with mRCC did not initiate systemic therapy. Although the underlying reasons were not granularly assessed per patient, common reasons include patients who were treated an AS approach, whereas others may have been too unwell for systemic therapy.

In a rapidly evolving landscape, there had been a lack of prospective phase III trials to guide the best approach to subsequent treatment after progression on contemporary first-line ICI-based combination therapy. Several retrospective studies suggest that VEGF-TKIs are clinically active after failure of ICI-based treatment.^24-28^ The VEGF-TKIs approved in first line (sunitinib and pazopanib) are considered reasonable choices^9^ and both agents are funded in Canada after first-line ipilimumab plus nivolumab.^29^ Cabozantinib is also considered a suitable option for subsequent therapy, after a subgroup analysis of the METEOR study suggested that it retained clinical activity after previous VEGF-TKI and anti–PD-(L)1 therapy.^30^ In an interim analysis, the recent phase II CaboPoint trial also showed that cabozantinib retained activity after treatment with ICI-based combination therapy.^31^ Cabozantinib first received Health Canada approval in 2018 for patients who had previous VEGF-TKI, and was subsequently approved after failure of VEGF-TKI and ICI. Cabozantinib is currently the only funded subsequent-line option after IO/TKI and it is also reimbursed as third-line therapy after progression on IO/IO and subsequent VEGF-TKI.^29^ For patients treated with upfront single-agent VEGF-TKI (sunitinib or pazopanib), axitinib, cabozantinib, and nivolumab are all reimbursed options for second-line treatment. Third-line therapy with either cabozantinib or nivolumab, depending on previous therapy, is also publicly funded in this group of patients.^29^ Lenvatinib plus everolimus was approved by Health Canada in 2017 for subsequent treatment after progression on VEGF-TKI, after a small phase II study showed superior progression-free survival compared with everolimus alone.^32^ However, it is not currently publicly reimbursed and, outside of clinical trials, is only accessible through patient support programs from the respective drug companies.

These data should be interpreted within the context of study design. Although the CKCis captures approximately 20% of all Canadian patients with kidney cancer, the CKCis population has been shown to have similar patient and tumor characteristics and reflects the wider national kidney cancer population.^11^ Patients in this study were all treated at academic centers and although our study does not directly capture treatments of patients in community cancer centers specifically, nor specific territorial variations, the referral of patients with mRCC largely occurs at CKCis centers, thus reflecting the wider consistencies in treatment utilization and sequencing. Although not a limitation of this work, tailored treatment for non–clear cell RCC remains an area of unmet need. Current guidelines suggest that in the absence of clinical trials, standard first-line therapies for clear cell RCC can be extrapolated to the non–clear cell setting.^9^ Although clinical outcomes were not the focus of this study, a preliminary analysis shows a higher objective response rate in cohort 2 compared with cohort 1 (36.9% *v* 23.7%; odds ratio, 0.58 [95% CI, 0.48 to 0.70]). Similarly, descriptive analysis of OS appears improved in cohort 2 compared with cohort 1 (median OS, 47.5 *v* 33.1 months). Future CKCis analyses are ongoing and will granularly describe clinical outcomes of immune-related therapies in the contemporary era.

In conclusion, the process of drug approval in a publicly funded health care system may be complex and can take longer than 1 year before new, life-prolonging therapies become publicly reimbursed. In this context, these real-world data provide reassuring evidence that utilization of systemic therapies for mRCC in Canada reflect global patterns of approval. Regional differences in drug reimbursement processes should be analyzed to understand their impact on patient clinical and quality-of-life outcomes. Equitable access for patients requires significant efforts leveraging government-funded clinical trials and access programs. Our work highlights the importance of ongoing advocacy to facilitate patient access to novel and efficacious oncology drug therapies.

## References

[1] Bukavina L Bensalah K Bray F, et al: Epidemiology of renal cell carcinoma: 2022 update. Eur Urol 82:529-542, 2022 36100483 10.1016/j.eururo.2022.08.019

[2] Brenner DR Poirier A Woods RR, et al: Projected estimates of cancer in Canada in 2022. CMAJ 194:E601-E607, 2022 35500919 10.1503/cmaj.212097 PMC9067380

[3] Lalani AKA McGregor BA Albiges L, et al: Systemic treatment of metastatic clear cell renal cell carcinoma in 2018: Current paradigms, use of immunotherapy, and future directions. Eur Urol 75:100-110, 2019 30327274 10.1016/j.eururo.2018.10.010

[4] Motzer RJ Tannir NM McDermott DF, et al: Nivolumab plus ipilimumab versus sunitinib in advanced renal-cell carcinoma. N Engl J Med 378:1277-1290, 2018 29562145 10.1056/NEJMoa1712126 PMC5972549

[5] Rini BI Plimack ER Stus V, et al: Pembrolizumab plus axitinib versus sunitinib for advanced renal-cell carcinoma. N Engl J Med 380:1116-1127, 2019 30779529 10.1056/NEJMoa1816714

[6] Motzer R Alekseev B Rha SY, et al: Lenvatinib plus pembrolizumab or everolimus for advanced renal cell carcinoma. N Engl J Med 384:1289-1300, 2021 33616314 10.1056/NEJMoa2035716

[7] Choueiri TK Powles T Burotto M, et al: Nivolumab plus cabozantinib versus sunitinib for advanced renal-cell carcinoma. N Engl J Med 384:829-841, 2021 33657295 10.1056/NEJMoa2026982 PMC8436591

[8] Only immuno-oncology combination therapy approved by health canada as first-line treatment for advanced or metastatic renal cell carcinoma: Cision. https://www.newswire.ca/news-releases/only-immuno-oncology-combination-therapy-approved-by-health-canada-as-first-line-treatment-for-advanced-or-metastatic-renal-cell-carcinoma-687680361.html

[9] Canil C Kapoor A Basappa NS, et al: Management of advanced kidney cancer: Kidney Cancer Research Network of Canada (KCRNC) consensus update 2021. Can Urol Assoc J 15:84-97, 2021 33830005 10.5489/cuaj.7245 PMC8021420

[10] Jackson EB Hotte SJ: Disparity in public funding of systemic therapy for metastatic renal cell carcinoma in Canada. Can Urol Assoc J 16:E516-E522, 2022 35704937 10.5489/cuaj.7846 PMC9665313

[11] Tajzler C Tanguay S Mallick R, et al: Determining generalizability of the Canadian Kidney Cancer Information System (CKCis) to the entire Canadian kidney cancer population. Can Urol Assoc J 14:E499-E506, 2020 33275557 10.5489/cuaj.6716 PMC7716824

[12] Heng DY Xie W Regan MM, et al: Prognostic factors for overall survival in patients with metastatic renal cell carcinoma treated with vascular endothelial growth factor-targeted agents: Results from a large, multicenter study. J Clin Oncol 27:5794-5799, 2009 19826129 10.1200/JCO.2008.21.4809

[13] Health Canada approves Opdivo (Nivolumab) for the treatment of advanced or metastatic renal cell carcinoma Bristol-Myers Squibb Canada Co, 2016. https://www.bms.com/ca/en/media/press-release-listing/2016-04-25-press-release.html

[14] Motzer RJ Escudier B McDermott DF, et al: Nivolumab versus everolimus in advanced renal-cell carcinoma. N Engl J Med 373:1803-1813, 2015 26406148 10.1056/NEJMoa1510665 PMC5719487

[15] SankeyMATIC. https://sankeymatic.com

[16] Gotfrit J Dempster W Chambers J, et al: The pathway for new cancer drug access in Canada. Curr Oncol 29:455-464, 2022 35200541 10.3390/curroncol29020041 PMC8870298

[17] CADTH reimbursement reviews process in brief. https://www.cadth.ca/cadth-reimbursement-reviews-process-brief

[18] Procedures for the CADTH pan-Canadian Oncology Drug Review. https://www.cadth.ca/sites/default/files/pcodr/pCODR%27s%20Drug%20Review%20Process/pcodr-procedures.pdf

[19] Albiges L Tannir NM Burotto M, et al: Nivolumab plus ipilimumab versus sunitinib for first-line treatment of advanced renal cell carcinoma: Extended 4-year follow-up of the phase III CheckMate 214 trial. ESMO Open 5:e001079, 2020 33246931 10.1136/esmoopen-2020-001079 PMC7703447

[20] Rini BI Plimack ER Stus V, et al: Pembrolizumab (pembro) plus axitinib (axi) versus sunitinib as first-line therapy for advanced clear cell renal cell carcinoma (ccRCC): Results from 42-month follow-up of KEYNOTE-426. J Clin Oncol 39, 2021 (suppl 15; abstr 4500)

[21] Porta C Eto M Motzer RJ, et al: Updated efficacy of lenvatinib (LEN) + pembrolizumab (PEMBRO) vs sunitinib (SUN) in patients (pts) with advanced renal cell carcinoma (aRCC) in the CLEAR study. Ann Oncol 33:S660-S680, 2022

[22] Powles T Choueiri TK Burotto M, et al: Final overall survival analysis and organ-specific target lesion assessments with two-year follow-up in CheckMate 9ER: Nivolumab plus cabozantinib versus sunitinib for patients with advanced renal cell carcinoma. J Clin Oncol 40, 2022 (suppl 6; abstr 350)

[23] Kushnir I Basappa NS Ghosh S, et al: Active surveillance in metastatic renal cell carcinoma: Results from the Canadian Kidney Cancer information system. Clin Genitourin Cancer 19:521-530, 2021 34158246 10.1016/j.clgc.2021.05.004

[24] Albiges L Fay AP Xie W, et al: Efficacy of targeted therapies after PD-1/PD-L1 blockade in metastatic renal cell carcinoma. Eur J Cancer 51:2580-2586, 2015 26346135 10.1016/j.ejca.2015.08.017

[25] Auvray M Auclin E Barthelemy P, et al: Second-line targeted therapies after nivolumab-ipilimumab failure in metastatic renal cell carcinoma. Eur J Cancer 108:33-40, 2019 30616146 10.1016/j.ejca.2018.11.031

[26] Barata PC De Liano AG Mendiratta P, et al: The efficacy of VEGFR TKI therapy after progression on immune combination therapy in metastatic renal cell carcinoma. Br J Cancer 119:160-163, 2018 29795307 10.1038/s41416-018-0104-z PMC6048048

[27] Shah AY Kotecha RR Lemke EA, et al: Outcomes of patients with metastatic clear-cell renal cell carcinoma treated with second-line VEGFR-TKI after first-line immune checkpoint inhibitors. Eur J Cancer 114:67-75, 2019 31075726 10.1016/j.ejca.2019.04.003 PMC7537491

[28] Wells JC Dudani S Gan CL, et al: Clinical effectiveness of second-line sunitinib following immuno-oncology therapy in patients with metastatic renal cell carcinoma: A real-world study. Clin Genitourin Cancer 19:354-361, 2021 33863648 10.1016/j.clgc.2021.03.006

[29] CADTH: Provisional Funding Algorithm. Renal cell carcinoma, 2023. https://www.cadth.ca/sites/default/files/pdf/PH0019-Adjuvant%20RCC-CAPCA%20Endorsement.pdf

[30] Choueiri TK Escudier B Powles T, et al: Cabozantinib versus everolimus in advanced renal cell carcinoma (METEOR): Final results from a randomised, open-label, phase 3 trial. Lancet Oncol 17:917-927, 2016 27279544 10.1016/S1470-2045(16)30107-3

[31] Albiges L Powles T Sharma A, et al: CaboPoint: Interim results from a phase 2 study of cabozantinib after checkpoint inhibitor (CPI) therapy in patients with advanced renal cell carcinoma (RCC). J Clin Oncol 41, 2023 (suppl 6; abstr 606)

[32] Motzer RJ Hutson TE Glen H, et al: Lenvatinib, everolimus, and the combination in patients with metastatic renal cell carcinoma: A randomised, phase 2, open-label, multicentre trial. Lancet Oncol 16:1473-1482, 2015 26482279 10.1016/S1470-2045(15)00290-9

